# Evaluation of Ex Vivo Adrenocorticotropic Hormone Responsiveness of Human Fetal Testis

**DOI:** 10.1210/endocr/bqad165

**Published:** 2023-11-03

**Authors:** Mariska A M Schröder, David Greenald, Renate Lodewijk, Antonius E van Herwaarden, Paul N Span, Fred C G J Sweep, Rod T Mitchell, Hedi L Claahsen-van der Grinten

**Affiliations:** Department of Pediatrics, Radboud Amalia Children's Hospital, Radboud University Medical Center, 6500 HB Nijmegen, The Netherlands; Department of Laboratory Medicine, Radboudumc Graduate School, Radboud University Medical Center, 6500 HB Nijmegen, The Netherlands; MRC Centre for Reproductive Health, Institute for Regeneration and Repair, The University of Edinburgh, and the Royal Hospital for Children and Young People, Edinburgh EH16 4TJ, UK; MRC Centre for Reproductive Health, Institute for Regeneration and Repair, The University of Edinburgh, and the Royal Hospital for Children and Young People, Edinburgh EH16 4TJ, UK; Department of Laboratory Medicine, Radboudumc Graduate School, Radboud University Medical Center, 6500 HB Nijmegen, The Netherlands; Department of Laboratory Medicine, Radboudumc Graduate School, Radboud University Medical Center, 6500 HB Nijmegen, The Netherlands; Department of Radiation Oncology, Radiotherapy & OncoImmunology Laboratory, Radboudumc Graduate School, Radboud University Medical Center, 6500 HB Nijmegen, The Netherlands; Department of Laboratory Medicine, Radboudumc Graduate School, Radboud University Medical Center, 6500 HB Nijmegen, The Netherlands; MRC Centre for Reproductive Health, Institute for Regeneration and Repair, The University of Edinburgh, and the Royal Hospital for Children and Young People, Edinburgh EH16 4TJ, UK; Department of Pediatrics, Radboud Amalia Children's Hospital, Radboud University Medical Center, 6500 HB Nijmegen, The Netherlands

**Keywords:** adrenocorticotropic hormone, human fetal testis, congenital adrenal hyperplasia, testicular adrenal rest tumors

## Abstract

Testicular adrenal rest tumors (TARTs), commonly occurring in males with congenital adrenal hyperplasia, may arise from chronic stimulation of adrenocorticotropic hormone (ACTH)–sensitive cells in the testes. It is not yet established whether the human fetal testis (HFT) is responsive to ACTH. To investigate this, we cultured HFT tissue with and without ACTH for up to 5 days, and quantified adrenal steroid hormones and expression of adrenal steroidogenic enzymes. Fetal testis and adrenal tissue produced high levels of testosterone and cortisol, respectively, indicating viability. In contrast to fetal adrenal tissues, the expression of ACTH receptor *MC2R* was either absent or expressed at extremely low levels in ex vivo HFT tissue and no clear response to ACTH in gene expression or steroid hormone production was observed. Altogether, this study suggests that the HFT is unresponsive to ACTH, which would indicate that a TART does not arise from fetal testicular cells chronically exposed to ACTH in utero.

Testicular adrenal rest tumors (TARTs) are a common complication in congenital adrenal hyperplasia (CAH) (1). Elevated adrenocorticotropic hormone (ACTH) levels due to suboptimal hormonal control are considered an important factor in the development of TART (1). TART may arise from fetal cells within the testis after chronic stimulation with high levels of ACTH. Indeed, cells within the murine fetal and neonatal testis (2, 3) express the ACTH receptor *MC2R* and are responsive to ACTH in vitro (2, 4, 5). Upon ACTH stimulation, fetal and neonatal mouse testes express the adrenal steroidogenic enzymes *CYP11B1* and *CYP21A1*, and increase the production of testosterone (2, 4, 5). The adult murine Leydig cells do not express MC2R (6). It is unclear whether the human fetal testis (HFT) is responsive to ACTH and could exhibit adrenal-like features. The expression of *MC2R* by the HFT is contested (7, 8). Here, we aimed to assess the ACTH responsiveness of HFT. Identification of ACTH responsive cells in the HFT will strengthen the theory that TART arises from an ACTH-sensitive cell population in the testis during fetal development. By understanding how these benign tumors develop, we can optimize and more precisely tailor treatment of males with CAH to prevent TART development.

## Materials and Methods

### Ex Vivo Tissue Culture

Steroid hormone production and steroidogenic enzyme expression by ex vivo cultured HFT with and without ACTH were quantified. Human fetal adrenal (HFA) tissue was included as positive control (9).

First- (9 postconception weeks [pcw], n = 1) and second-trimester (13-14 pcw, n = 5) fetal testes and adrenals were collected following elective surgical termination of pregnancy. Ethics approval was obtained from the Lothian Research Ethics Committee (LREC08/H0712/3+5; LREC08/H0906/21+5). Fetal age was determined by scanning crown–rump length and foot length. Tissues were kept at 4 °C until processing. Male sex of the fetus was confirmed by quantitative polymerase chain reaction of the *SRY* gene (Methods (10)).

HFT and HFA derived from the same fetus (n = 6) were dissected and cut into ∼1 mm^3^ fragments. Fragments were cultured in 40-µL hanging drops of basal culture media (MEMα medium [Lonza] supplemented with 1× MEM Non-essential Amino Acids [Sigma], 1 × insulin transferrin and selenium [Sigma], 1 mM sodium pyruvate [Gibco], 2 mM L-glutamine, penicillin/streptomycin, and 10% fetal bovine serum) (9, 11). After 24 hours, fragments were randomly assigned to either 10 nM ACTH 1-24 (Tetracosactide; ALFASIGMA) or basal media culture. The ACTH concentration was based on previous ex vivo human cell and tissue culture studies (9-14). Adrenal and testis tissue fragments from 2 biological replicates (both 14 pcw) were also exposed to 100 and 1000 nM ACTH. A minimum of 2 available fragments per experimental condition was used. Fetal adrenal and testis tissue fragments were cultured for an additional 4 days at 37 °C under 5% CO_2_ with treatment/media change every 48 hours. Conditioned culture media collected from the ex vivo culture experiments were pooled for all technical replicates at each time-point per experimental condition (ACTH or basal media). After 5 days of culture, RNA was either isolated directly or tissue was snap frozen in liquid nitrogen and stored at −80 °C until RNA isolation (n = 5). Additionally, HFA and HFT fragments from a 14 pcw fetus were collected and fixed either prior or after culture and stained with hematoxylin and eosin for histological evaluation. Baseline *MC2R* expression (prior to culture) was assessed using RNA isolated from 3 additional cryopreserved HFT tissues (9, 13, and 14 pcw) and 3 cryopreserved HFA tissue samples (from 2 adrenal samples also used in culture (9 and 13 pcw), and 1 additional HFA tissue sample (14 pcw)).

### Gene Expression

Baseline or cultured tissue fragments were lysed in RLT buffer (Qiagen) with 10% β-mercaptoethanol, using a TissueLyser LT (Qiagen). RNA was isolated using the RNeasy-micro-plus kit (Qiagen) and reversed transcribed using SuperScript IV First-Strand Synthesis System (Invitrogen) with random hexamers in a Biometra Thermocycler (5 minutes at 65 °C). *CYP21A2*, *CYP11B1*, and *MC2R* cDNA levels were quantified as described (15), normalized to the geometric mean expression of the housekeeping genes GAPDH and PBGD, and presented relative to the average adrenal expression in basal media conditions. The expression was set to 0 when Ct>40. For Ct values above 32, expression was regarded as the detection limit.

### Quantification of Steroid Hormone Levels in Culture Media

Steroid hormone levels (cortisol, 21-deoxycortisol, 11-deoxycortisol, androstenedione, testosterone, 17-hydroxyprogesterone, and progesterone) from conditioned culture media were quantified using liquid chromatography tandem mass spectrometry (Methods (10)). Steroid levels in conditioned media collected after initial (unexposed) 24 hours culture were used to normalize steroid hormone levels in conditioned media quantified after 2 or 4 days of treatment, in order to normalize for variability in fragment size and percentage of steroid-producing cells.

### Statistical Analyses

Expression data are presented as mean with SE and steroid data as median with full range. The Wilcoxon signed-rank test was used to examine the effect of ACTH exposure on steroid hormone levels in the culture media, including biological replicates as paired observations. *P* < .05 was considered to be statistically significant.

## Results

### Morphology of Ex Vivo Cultured Human Fetal Testis and Adrenal

There was no observable difference in morphology between 10 nM ACTH-exposed fragments and fragments cultured under basal conditions (Fig. S1A (10)).

### Gene Expression

**Figure 1.**
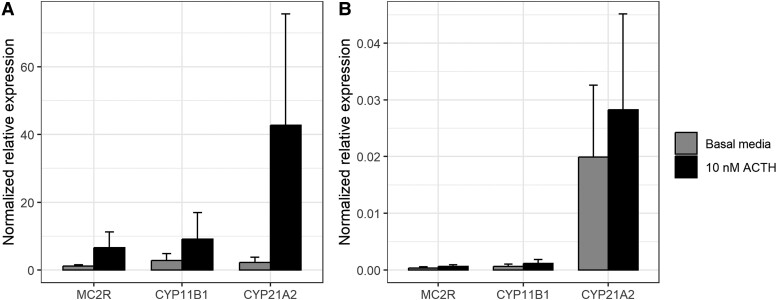
Normalized relative expression (mean ± SE) of *CYP11B1*, *CYP21A2*, and *MC2R* by ex vivo cultured fetal adrenal fragments (A) and fetal testis fragments (B), cultured for 5 days and exposed for 4 days to 10 µM ACTH (black) or basal media control (gray). Differences in expression between the ACTH-exposed and unexposed condition were tested using a paired t-test (n = 5).

*MC2R* was expressed by HFA tissue (cycle threshold [Ct] ranged from 21.3 to 23.8 in basal condition), but was at the detection limit in HFT tissue (Ct ranged from 32.9 to undetectable [>40] in basal condition). To eliminate the possibility that *MC2R* expression rapidly disappears in culture, *MC2R* expression was also assessed at baseline. While HFA clearly exhibited *MC2R* expression (Ct ranged from 19.9 to 21.6), Ct values were consistently low (ranging between 29.6 and 31.3) for HFT, resulting in a normalized relative expression of *MC2R* that was over 1000-fold lower in HFT compared with the HFA tissue with lowest *MC2R* expression. *CYP11B1* and *CYP21A2* were expressed by all HFA tissues. Exposure to 10 nM ACTH increased the expression of *CYP21A2* (19-fold, *P* = .29, df = 4, paired t-test) and *MC2R* (5.5-fold; *P* = .30), although statistical significance could not be established (Fig. 1A).

*CYP11B1* and *CYP21A2* expression was substantially lower in HFT than in HFA (Fig. 1). *CYP21A2* was expressed in HFT (Ct ranged from 28.8 to 34.0) but the expression of *CYP11B1* by HFT ranged from borderline detectable to undetectable (Fig. 1B; Ct 31.8->40). Exposure to 10 nM ACTH did not affect *CYP21A2* nor *CYP11B1* expression by HFT.

Thus, MC2R expression by the HFT is not evident and no clear response to ACTH in *CYP11B1* or *CYP21A2* expression by the HFT was observed during 4 days of exposure.

### Steroid Hormone Production

Steroids were detected in culture media of ex vivo cultured HFA and HFT fragments, confirming tissue viability and functional activity. A decline in steroid production over time was observed (Table S1 (10)). HFT and HFA produced high levels of testosterone or cortisol, respectively (Table S1 (10)). Progesterone and 21-deoxycortisol levels could not be detected (<0.625 nmol/L and <1.75 nmol/L) in most samples (data not shown).

Due to variability in fragment size and cell composition, some differences were observed in steroid hormone production at baseline between the assigned experimental conditions (Table S1 (10)). To control for this, levels were normalized for hormone levels quantified after the 24 hours initial culture period (Table S2 (10)).

**Figure 2.**
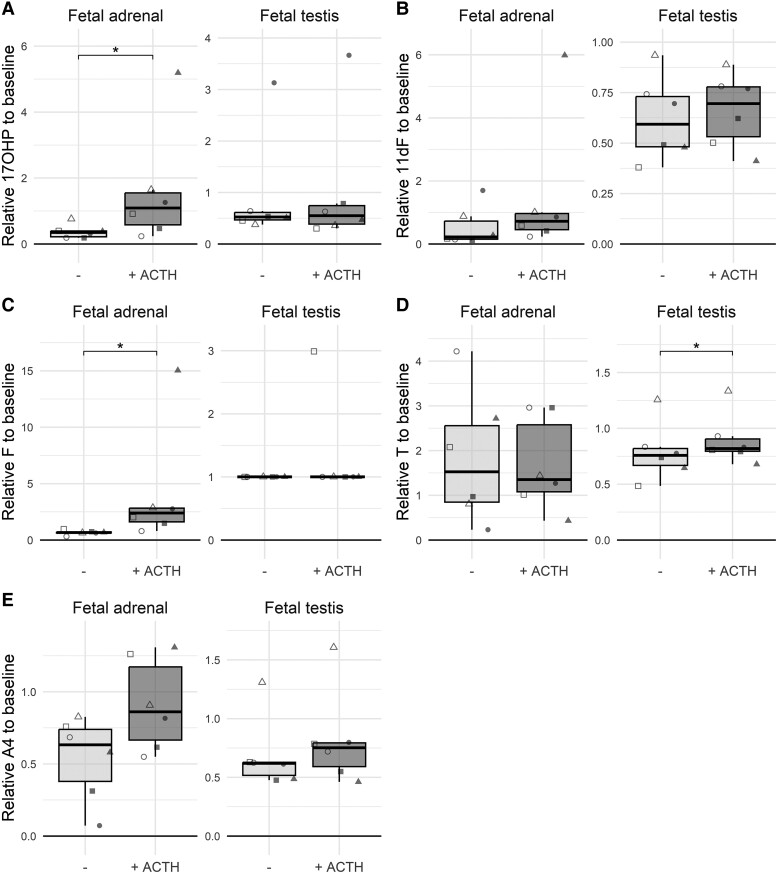
Normalized levels of (A) 17-hydroxyprogesterone (17OHP), (B) 11-deoxycortisol (11dF), (C) cortisol (F), (D) testosterone (T), and (E) androstenedione (A4) in culture media collected after 48 hours of ex vivo culture of HFA tissue fragments and HFT tissue fragments with or without exposure to 10 nM ACTH. Steroid hormone levels were normalized for steroid hormone levels in culture media collected after a preceding 24-hour basal culture period without ACTH exposure. Data of individual biological replicates (n = 6) are presented with unique symbols.

After 48-hour ACTH treatment, HFA fragments produced more 17-hydroxyprogesterone (2.4-fold) and cortisol (3.5-fold) compared with basal levels, while 10 nM ACTH did not (significantly) increase 11-deoxycortisol, androstenedione, or testosterone production by HFA (Fig. 2).

Testosterone, 17-hydroxyprogesterone, 11-deoxycortisol, and androstenedione could be quantified in HFT media (Table S1 (10)), but cortisol was undetectable. Exposure to 10 nM ACTH for 48 hours did not affect 17-hydroxyprogesterone, 11-deoxycortisol, or androstenedione levels (Fig. 2), and while statistically significantly higher levels of testosterone were found, the ratio was only 1.07 (Table S2 (10)).

To assess the response to prolonged exposure to ACTH, tissues were cultured for an additional 48-hour period (96 hour time-point) with or without ACTH (Fig. S2 (10)). The effect on testosterone production by HFT tissue was no longer observed. However, the stimulating effect of 10 nM ACTH on 17-hydroxyprogesterone, 11-deoxycortisol, and cortisol production by HFA tissue was even more pronounced (Fig. S2 (10)).

Exposure to increased doses (100 or 1000 nM) of ACTH did not indicate effects on expression of *CYP21A2* or *CYP11B1* or steroid hormone production by HFT after 48 hours or 96 hours (n = 2; data not shown).

## Discussion

To investigate if steroidogenic cells in healthy human fetal testes are responsive to ACTH, as hypothesized from mice studies, we examined the effect of ACTH on human fetal tissues. Unexpectedly, this study did not find a response to ACTH by the HFT, despite the tissues producing high levels of steroid hormones, indicating that they remained functionally active ex vivo. Fetal adrenal tissue showed a clear response to ACTH, validating our assay.

MC2R expression was either absent or expressed at very low levels in HFT, confirming earlier studies (7) and our previous exploration (15) of publicly available single cell RNA-sequencing data of HFT (GSE143356 (16)). O'Shaughnessy et al (8) reported detectable MC2R, CYP11B1, and CYP21 expression by second trimester HFT. However, MC2R was not detected in all fetal testes in their study and no reference gene was used. Therefore, MC2R expression may be negligible in these tissues.

Remarkably, 11-deoxycortisol was produced by human fetal testes, independent of ACTH exposure. CYP21A2, which is required to produce 11-deoxycortisol, was expressed at low levels by HFT. Further investigation of available single cell RNA-sequencing data of HFT could facilitate the exploration of adrenal-like features of fetal testis tissue.

Sample size was an important limitation in our study, which may have influenced our findings. An additional limitation is the variability in steroid hormone production in response to ACTH observed between cultured tissue fragments. However, this variability is expected (8, 9) and consistent with previous observations of inter-fetus variability by the HFA (9). Gonadotropin-responsiveness of our cultured human fetal testes (positive control) could not be validated in parallel with the ACTH exposure and basal media conditions, due to the limited number of fragments obtained from human fetal testes, preventing the inclusion of more than 2 experimental conditions (ACTH and basal media). Although a limitation, the high levels of testosterone secreted by HFT into culture media confirm that the cultured HFT tissues were functionally active.

Contrary to the situation in mice, this study suggests that the HFT is unresponsive to ACTH. Further research, including larger sample sizes, is required to validate this finding.

## Data Availability

Original data generated and analyzed during this study are included in this published article or are deposited into data repositories listed in References.

## Abbreviations

ACTH: adrenocorticotropic hormone
CAH: congenital adrenal hyperplasia
Ct: cycle threshold
HFA: human fetal adrenal
HFT: human fetal testis
PCW: postconception weeks
TART: testicular adrenal rest tumor
